# Exploring Autologous Dendritic Cells for T Cell Modulation: A Step Towards Personalized Medicine in Leishmaniasis

**DOI:** 10.3390/cells15100919

**Published:** 2026-05-18

**Authors:** Mafalda Meunier, Ana Valério-Bolas, Armanda Rodrigues, Flávia Fróis-Martins, Rui Ferreira, Inês Cardoso, Marta Monteiro, Joana Palma-Marques, Manuela Carvalheiro, Telmo Nunes, Wilson T. Antunes, Graça Alexandre-Pires, Isabel Pereira da Fonseca, Gabriela Santos-Gomes

**Affiliations:** 1 Global Health and Tropical Medicine (GHTM), LA-REAL, Instituto de Higiene e Medicina Tropical (IHMT), Universidade NOVA de Lisboa, 1349-008 Lisboa, Portugal; mmeunier@ihmt.unl.pt (M.M.); ana.bolas@ihmt.unl.pt (A.V.-B.); joana.palma.marques@gmail.com (J.P.-M.);; 2 Banco de Sangue Animal (BSA), 4100-462 Porto, Portugal; 3 Centre for Interdisciplinary Research in Animal Health (CIISA), Faculty of Veterinary Medicine, University of Lisbon, 1300-477 Lisbon, Portugalgpires@fmv.ulisboa.pt (G.A.-P.); ifonseca@fmv.ulisboa.pt (I.P.d.F.); 4 Associate Laboratory for Animal and Veterinary Sciences (AL4AnimalS), 1300-477 Lisboa, Portugal; 5 Research Institute for Medicines (iMed), Faculdade de Farmácia, Universidade de Lisboa, 1649-003 Lisboa, Portugal; mcarvalheiro@ff.ulisboa.pt; 6 Microscopy Center, Faculty of Sciences of the University of Lisbon-FCUL-BioISI Ce3CE, 1749-016 Lisboa, Portugal; 7 Instituto Universitário Militar (IUM), Centro de Investigação, Desenvolvimento e Inovação da Academia Militar (CINAMIL), Unidade Militar Laboratorial de Defesa Biológica e Química (UMLDBQ), 1100-471 Lisboa, Portugal; antunes.wdt@mail.exercito.pt

**Keywords:** *Leishmania*, extracellular vesicles, lymphocytes, memory T cells, innate receptors, immune mediators

## Abstract

**Highlights:**

1. Dendritic cells primed with *Leishmania* extracellular vesicles modulate acquired immune responses.
2. Dendritic cells promoting a balanced pro- and anti-inflammatory T cell response.
3. This cell modulatory approach represents a promising first step toward personalized immunotherapy for Leishmaniasis.

**Abstract:**

Leishmaniasis caused by *Leishmania infantum* is a zoonotic disease endemic in many regions worldwide. The antigen-presenting dendritic cells (DCs) bridge the innate and adaptive immune response by activating T lymphocytes. Therefore, the present study examines whether T lymphocyte activation can be directed by autologous DCs primed by extracellular vesicles (EVs) derived from *L. infantum*. For this, lymphocytes were co-cultured with monocyte-derived DCs (moDCs) that were primed by EVs. moDC signaling and activation were examined by gene expression of toll-like receptors and cytokines. The antigen-presentation ability was analyzed through major histocompatibility complex molecules, and T cell subpopulations were explored by immunophenotyping. In co-cultures, EV-primed moDCs upregulated TLR2, TLR4, and TLR9, along with overexpression of MHC molecules. Co-cultures involving moDCs primed by EVs promoted the upregulation of both pro-inflammatory and regulatory cytokines associated with the expansion of non-conventional regulatory and central memory T cell subsets within the CD8^+^ T cell subpopulation. These findings suggest that activated moDCs can modulate cytotoxic lymphocytes, thereby promoting a balanced inflammatory microenvironment counterbalanced by a concurrent regulatory immune response. Thus, cell-based immune strategies using moDCs loaded with *Leishmania*-derived EVs represent a potential first step toward the development of innovative and personalized immune prophylactic and therapeutic approaches for leishmaniasis.

## 1. Introduction

Leishmaniasis is considered a neglected tropical disease since it affects mostly impoverished and marginalized communities while leading to devastating health, social, and economic burdens [1]. According to the World Health Organization [2], more than 20 species of *Leishmania* can cause disease in mammals, including canids, rodents, marsupials, edentates, and primates [3]. In humans, *Leishmania* spp. can cause distinctive clinical forms of leishmaniasis, and the number of new leishmaniasis cases can reach up to a million per year [2]. However, the spread of *Leishmania* is increasing, primarily due to epidemiological alterations, such as global climate change associated with the expansion of sandflies to other areas, which are contributing to the emergence of this parasitic infection [4,5,6].

Domestic dogs represent the principal reservoir host of *L. infantum*, which can cause canine leishmaniasis (CanL) and zoonotic visceral leishmaniasis in humans. CanL is endemic throughout different regions of the world, i.e., the Mediterranean Basin, Latin America, and Asia. The disease has been associated with poor rural and suburban areas [7] but over the years has also been observed in urban areas, due to environmental alterations, changes in the ecology and biology of the vector, and migration from rural areas to urban centers [8,9]. In Southwestern Europe, it was estimated that 2.5 million dogs could be infected with *L. infantum*, and the disease is spreading northward due to sandfly dispersal driven by climate change and the movement of dogs across borders [10,11].

The outcome of *L. infantum* infection depends on multiple factors, including age, breed, fur size, living conditions, genetic background, and the host’s immune competence. The process starts when an infected female phlebotomine sandfly takes a blood meal, biting the host’s skin and releasing parasites into the dermis. This triggers endothelial activation that, through the release of chemokines, activates phagocytic cells, including dendritic cells (DCs). DCs are antigen-presenting cells (APCs) that can internalize the parasite. This is followed by the processing of parasite peptides, cell maturation, and the expression of major histocompatibility complex (MHC) and co-stimulatory molecules on its surface. Afterward, loaded DCs migrate to the secondary lymphoid organs, where antigen presentation occurs to naïve T lymphocytes [12]. This involves small parasite peptides complexed with MHC class I (MHCI) or class II (MHCII) molecules, which can target T cell receptors (TCRs), thereby inducing an adaptive immune response [13]. Furthermore, mature DCs can synthesize pro-inflammatory immune mediators that can promote the activation of other innate immune cells and the polarization of naïve T cells into type 1 T helper cells (Th1). In turn, the release of pro-inflammatory immune mediators can promote a positive feedback loop for DCs. However, *Leishmania* parasites have developed ingenious ways to hide from host immune recognition, evading the immune response and modulating immune activation by subverting DC signaling and downregulating gene expression, which can ensure the successful establishment of infection [14,15,16,17,18,19]. It has been reported that *L. infantum*-infected DCs exhibited an immature phenotype, reduced activation, impaired migration, and diminished antigen presentation capacity [20]. In addition, *Leishmania* extracellular vesicles (EVs), which are lipid-bound nanovesicles shed by eukaryotic cells into the extracellular space, can establish intracellular communication with the host cells [19,21,22], thereby modulating immune response. It was reported that *L. donovani*-derived EVs failed to prime monocyte-derived DCs (moDCs) and subsequently avoided the differentiation of naive CD4^+^ T cells into interferon (IFN)-γ-producing Th1 cells. Moreover, EVs from *L. amazonensis* can modulate canine moDCs to promote activation of CD8^+^ T cells, and *L. major*-derived EVs favor Th2 polarization and exacerbation of disease [22,23]. Other authors have also demonstrated that EVs shed by *Leishmania* promastigotes can modulate both innate and adaptive immune responses, triggering inflammation [24,25]. Therefore, it has been recognized that EVs can be a powerful immunomodulatory tool with the potential to coordinate the host immune response against *Leishmania* infection.

During infection and upon stimulation by APCs, naïve T cells undergo vigorous proliferation. While most of these activated T cells undergo apoptosis, a small fraction differentiates into long-lived memory T cells. Upon reinfection with the same pathogen, these memory T cells differentiate into effector T cells, boosting a faster and more effective immune response [26,27]. Two key molecules, CD44 and CD62L, have been implicated in the differentiation of naïve T cells into either activated or memory T cells. CD44 is highly upregulated following lymphocyte activation and remains highly expressed in memory T cells. In contrast, CD62L, a L-selectin adhesion molecule that plays a role in directing lymphocyte homing to secondary lymphoid compartments, can be downregulated. Thus, naïve T cells typically exhibit a CD44^−^CD62L^+^ phenotype [28]. As these cells differentiate into memory cells, central memory T cells (T_CM_) acquire a CD44^+^CD62L^+^ profile while effector memory T cells (T_EM_) present a CD44^+^CD62L^−^ molecular phenotype. These memory cells lack molecular markers that direct them to lymph node homing and instead migrate to inflamed tissues, where they can survey the microenvironment. Upon re-encountering the assigned antigen, these cells can immediately differentiate into effector cells [26], enabling a quick and antigen-specific local immune response. The CD44^−^CD62L^−^ T cell subset represents an intermediate stage of T cells, in the transition of naïve to fully differentiated T_EM_ [29].

Therefore, the current study aims to analyze the ability of canine DCs, differentiated in vitro (moDCs) and exposed to *L. infantum* parasites or EVs, to direct the activity of autologous T lymphocytes by examining the gene expression of DCs’ innate receptors and the likely antigen presentation, the differentiation of T cell subpopulations, and the generation of immunomodulators.

## 2. Materials and Methods

### 2.1. Experimental Design

To evaluate the interplay of *L. infantum*-stimulated DCs and lymphocytes, peripheral blood mononuclear cells (PBMCs) were isolated from healthy dogs using a density gradient. Then, moDCs were differentiated from monocytes through a cocktail of immune mediators [23,24]. In vitro, moDCs were exposed to *L. infantum* promastigotes, or stimulated with parasite-soluble antigens (Ag) or *L. infantum*-derived EVs. Co-cultures of loaded moDCs and autologous lymphocytes isolated from PBMCs were established. The signalization of moDCs was analyzed by Toll-like receptors and cytokine gene expression through reverse transcription real-time quantitative PCR (RT-qPCR). T cell subpopulations (helper, cytotoxic, regulatory, and memory) and moDCs expressing MHCI or MHCII molecules at the cell surface were characterized phenotypically by multiparametric flow cytometry.

### 2.2. Blood Samples and Parasites

The sample size of six to ten healthy dogs’ blood samples was defined by a two-tailed paired *t*-test (α = 0.05), taking into consideration the number of IL-10 gene copies quantified by RT-qPCR. These dogs came from a natural population, which included dogs of both genders and different breeds, with ages ranging between 2 and 9 years old, weighing over 20 kg, and had not been vaccinated against CanL. They presented with a normal blood count and serum values. In all samples, serological tests were performed to detect anti-*Leishmania* antibodies using a rapid immunochromatographic test (SNAP^®^ *Leishmania*, IDEXX), with all results being negative. Afterward, confirmatory testing was performed by qPCR for *L. infantum, Anaplasma* spp., *Babesia canis*, *Dirofilaria immitis*, and *Ehrlichia* spp., presenting negative results in all cases. The use of canine blood samples in the current study was approved by the Ethics and Welfare Committee of the Faculty of Veterinary Medicine, University of Lisbon (Portugal), and conducted in accordance with institutional guidelines and in compliance with the European Community Council Directive of 24 November 1986. Informed consent was obtained from dog tutors, who were fully informed about the study objectives and the nature of the procedures.

*L. infantum* virulent promastigotes (MHOM/PT/89/IMT151) collected in the stationary phase of growth of axenic subcultures up to a maximum of five passages [30] were used to infect moDCs. Parasites were maintained in vitro in Schneider’s Drosophila medium (SCHN, Biowest, Nuaillé, France) supplemented with 10% (*v*/*v*) heat-inactivated fetal bovine serum (hiFBS, Biowest), penicillin-streptomycin (Pen-Strep, Sigma-Aldrich, Darmstadt, Germany) at 100 U.mL^−1^ and 100 μg.mL^−1^, respectively (complete SCHN medium), and incubated at 24 °C.

### 2.3. L. infantum Soluble Antigen

*L. infantum* soluble antigen (Ag) was obtained from high-density cultures of *L. infantum* promastigotes. Parasites were collected by centrifugation (1800× *g*, 15 min, room temperature). The supernatant was discarded, and the pellet was washed twice with 1× phosphate buffer saline (PBS, VWR) and centrifuged. The pellet was resuspended in 1× PBS. To lyse the parasites, six freeze–thaw cycles were performed, alternating between −20 °C and room temperature, and between each cycle suspensions were vortexed for 30 s. The solution was then centrifuged, and the supernatant was collected. The concentration of Ag was quantified using a Nanodrop 1000 Spectrophotometer (Thermo Fisher Scientific, Waltham, MA, USA) and adjusted to 40 μg.mL^−1^.

### 2.4. Purification of L. infantum-Derived Extracellular Vesicles

A high-density axenic culture of *L. infantum* promastigotes was centrifuged, and the pellet was resuspended in SCHN medium supplemented with 5% (*v*/*v*) hiFBS and 5% (*v*/*v*) exosome-depleted FBS (Thermo Fischer Scientific) for 72 h to accumulate EVs sheared by parasites. This allows for gradual adaptation of the parasites to the medium supplemented with exosome-depleted FBS, minimizing the interference of bovine exosomes in the study, which can cause significant background issues. Promastigotes were then washed and resuspended in SCHN medium supplemented with 10% (*v*/*v*) exosome-depleted FBS for 72 h.

Afterward, the parasite suspension was centrifuged to remove parasites, and the supernatant was collected and centrifuged again (3000× *g*, 30 min, 4 °C) to remove cellular debris. Thereafter, the total exosome isolation reagent (Invitrogen™) was added to the supernatant at 1:2 ratio (isolation reagent: supernatant), vortexed for 30 s, and incubated at 4 °C for 24 h. The solution was centrifuged (10,000× *g*, 1 h, 4 °C), and the pellet containing extracellular vesicles (EVs) resuspended in an appropriate volume of 1× PBS [24].

Although nanoparticle tracking analysis can be applied for the characterization of parasitic EVs, an alternative strategy has been used in the current study to ensure a comprehensive assessment of all EV subtypes shed by *L. infantum* promastigotes in axenic culture. Thus, two complementary approaches were applied to examine the size, morphology, and distribution of *L. infantum*-derived EVs. Scanning electron microscopy (SEM) enabled direct observation of EVs, while dynamic light scattering (DLS) was used to estimate their size distribution, minimizing potential bias associated with sample complexity due to the potential presence of co-precipitated proteins from the purification process. The topographical analysis of the *L. infantum*-derived EVs was performed by SEM, alongside viable promastigotes, as previously described [24]. The EVs were examined under an ultra-high-resolution scanning electron microscope (Hitachi SU8010, Hitachi High-Technologies Corporation, Chiyoda, Tokyo), and images were captured. The size distribution of *L. infantum*-derived EVs was generated based on the mean signal intensity (pixel intensity) using Image J 1.54g (Wayne Rasband and contributors, National Institutes of Health, Bethesda, MD, USA).

EVs were then analysed by DLS using Zetasizer Nano-S equipment (Malvern Instruments, Malvern, UK) at a constant temperature of 25 °C, with detection at a scattering angle of 90°. The zeta potential (ζ), which is an indicator of membrane charge and is an important indicator of EV stability, was determined by electrophoretic light scattering (ELS) at pH 7.5 using Zetasizer Nano-Z equipment (Malvern Instruments). A negative control for the isolation method, consisting of sterile Schneider’s medium supplemented with exo-free FBS and subjected to the same EV isolation procedure, was also analyzed by both DLS and ELS. Results are expressed as mean ± standard deviation from three independent isolates.

To characterize EVs further, their specific immunoreactivity was evaluated using a serological assay, which provides valuable information but does not replace comprehensive molecular marker profiling. However, it circumvents reliance on conventional molecular markers, which may exhibit biological variability and thereby limit the consistent detection of *L. infantum*-derived EVs. Furthermore, the molecular characterization of EVs, including the glycoprotein of 63 kDa or other molecular markers, has already been examined in previous proteomic studies [31,32]. Therefore, an in-house ELISA assay was developed to assess whether antibodies present in *L. infantum*-positive canine serum specifically recognize antigens associated with *L. infantum* EVs. As a positive control, *L. infantum* soluble antigen (Ag) [24] was included, together with the EV-isolation negative control. Briefly, 96-well plates were coated overnight at 4 °C with L. infantum EVs at 100 µg/mL in 100 µL of coating buffer (14.1 mM Na_2_CO_3_, 39.4 mM NaHCO_3_). Plates were washed three times with 0.05% Tween 20 in 1× PBS. Then, *L. infantum* positive canine serum (BioSystems Leishmania/Dog positive control Ref. 44953 Lot 003XA) was added at serial dilutions 1:80, 1:160, and 1:320 in a final volume of 100 µL per well and incubated for 1 h at 37 °C. After washing, the wells were blocked with blocking buffer (1% BSA in 1× PBS) for 1 h at 37 °C. Horseradish peroxidase (HRP)-conjugated goat anti-dog IgG (H+L) antibody was then added at a 1:5000 dilution and incubated for 1 h at 37 °C. Following a final series of washes, the reaction was developed using 3,3′,5,5′-tetramethylbenzidine (TMB, Sigma-Aldrich) substrate for 15 min at room temperature in the dark. The reaction was stopped with 0.2 M sulfuric acid, and absorbance was measured at 450 nm in a microplate reader (BIOTEK^®^ FLx800, BioTek Instruments GmbH, Luzern, Switzerland).

EV protein content was estimated in triplicate using a Nanodrop 1000 Spectrophotometer (Thermo Scientific, Waltham, MA, USA) at 280 nm, and the concentration was adjusted to 50 μg.mL^−1^.

### 2.5. Isolation of Lymphocytes and Monocytes and Differentiation into moDCs

To isolate PBMCs, whole blood samples were centrifuged to separate the buffy coat, which is rich in white blood cells. Using a density gradient method, PBMCs were isolated from buffy coats [33,34]. Histopaque-1077 (Sigma-Aldrich) was added to a Falcon tube, followed by the buffy coat at a 1:1 ratio, and centrifuged (340× *g*, 30 min, room temperature). Then, the PBMC layer located between the plasma and Histopaque was collected and resuspended with a 0.9% (*w*/*v*) sodium chloride (NaCl, VWR) solution, and then centrifuged (340× *g*, 10 min, room temperature). To lyse any remaining red blood cells, the pellet was resuspended in sterile ultrapure water and mixed with an equal volume of 1.8% (*w*/*v*) NaCl solution, followed by centrifugation. The pellet was washed in 1× PBS, and the cells were resuspended in Roswell Park Memorial Institute 1640 medium (RPMI, Sigma-Aldrich) supplemented with 10% (*v*/*v*) hiFBS and Pen-Strep (complete RPMI medium). The viability and concentration of PBMCs were assessed using trypan blue staining in a Neubauer chamber under an optical microscope.

**Figure 1:**
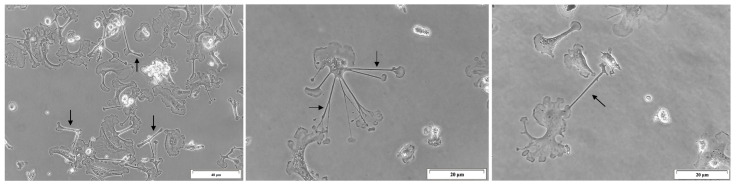
Morphology of moDCs. Images representative of moDCs with 7 days of differentiation were acquired under an optical inverted microscope at 200× and 400× magnification. Arrows indicate dendrites characteristic of DCs.

To differentiate moDCs in vitro, PBMC suspension at a concentration of 2.5 × 10^5^ cells.mL^−1^ was cultured in 24-well plates (VWR) with complete RPMI medium for 24 h at 37 °C in a humidified atmosphere containing 5% CO_2_, allowing the monocytes to adhere to the wells. Afterward, the supernatant containing lymphocytes and other non-adherent cells was collected and centrifuged (400× *g*, 10 min, room temperature). The pellet was then resuspended in 90% (*v*/*v*) hiFBS and 10% (*v*/*v*) dimethyl sulfoxide (DMSO, Sigma-Aldrich) and stored at −80 °C for later use. Complete RPMI medium supplemented with 10% (*v*/*v*) colony-stimulating factor (CSF) and 100 ng.mL^−1^ of canine recombinant interleukin (IL)-4 (crIL-4, R&D Systems) [33] was added to adherent cells and incubated at 37 °C in a humidified atmosphere containing 5% CO_2_. After 4 days, half of the medium was replaced with fresh medium. A total of 7 days of differentiation was required to obtain moDCs [20] (Figure 1).

The growth factor CSF that induces cell proliferation and differentiation [34,35] was obtained from the murine immortalized fibroblast L929 cell line.

### 2.6. Lymphocytes and moDC Co-Cultures

After 7 days of differentiation, the morphology of the moDCs was observed, and cells were counted under a CKX41 inverted microscope (Olympus, Hachioji, Tokyo). To reduce the amount of cellular stress or damage, the cells were not detached. Instead, assuming that moDCs were evenly distributed, cell concentration was estimated by counting ten random fields throughout different wells at 400× magnification. Knowing the area of each well, a direct proportionality was obtained between the cell count average and the area observed.

In 24-well plates (VWR), moDCs were incubated (37 °C, humidified atmosphere containing 5% CO_2_) for 24 h with virulent *L. infantum* promastigotes, at a 1:3 ratio (cell: parasite), Ag (40 μg.mL^−1^), *L. infantum*-derived EVs (50 μg.mL^−1^), or with phorbol myristate acetate (0.2 μg.mL^−1^ PMA, Promega, Madison, WI, USA) In parallel, unprimed moDCs were also incubated for 24 h. moDCs were then added to autologous lymphocytes at a 1:2 ratio (moDCs: lymphocytes) and incubated for 72 h. In parallel, lymphocytes stimulated with concanavalin A (5 μg.mL^−1^ ConA, Sigma-Aldrich) and resting lymphocytes were incubated for 72 h.

After 72 h of incubation, the levels of viable lymphocytes and moDCs exposed to *L. infantum* promastigotes or stimulated by Ag or EVs were evaluated by multiparametric flow cytometry. Unstimulated lymphocytes and unprimed moDCs, as well as lymphocytes and moDCs stimulated with ConA and PMA, respectively, were also analyzed, and the viability index was estimated. Cells were washed with 1× PBS (400× *g*, 10 min, 4 °C), incubated with a TACS^TM^ Annexin V-FITC Apoptosis Detection Kit (R&D Systems, Minneapolis, MN, USA), according to the manufacturer’s instructions, and then treated with 1 μL of propidium iodide (PI) (R&D Systems). Subsequently, cells were acquired by a flow cytometer (CytoFLEX, Beckman Coulter, Brea, CA, USA). A gating strategy using Annexin V versus PI was employed to distinguish between three different populations: Annexin V^−^PI^−^ (viable cells), Annexin V^+^PI^−^ (pre-apoptotic cells), and Annexin V^+^PI^+^ (apoptotic cells).

### 2.7. Scanning Electron Microscopy

Scanning electron microscopy (SEM) enables high-resolution observation of cell size, shape, and topography. The morphology and interaction of *L. infantum* with moDCs and lymphocytes were evaluated by SEM. Co-cultures of lymphocytes and moDCs previously stimulated with *L. infantum* promastigotes for 2 h and 4 h were analyzed.

Sample preparation was performed in sterile 13 mm round glass coverslips placed in a 24-well plate. Then, 3 × 10^5^ cells.mL^−1^ of moDCs were added to each well and incubated with *L. infantum* promastigotes at a 1:3 (cell: parasite) ratio for 2 h and 24 h at 37 °C in a humidified atmosphere containing 5% CO_2_. After incubation, coverslips were fixed with 1× PBS 2% (*w*/*v*) paraformaldehyde (Sigma-Aldrich, PBS-Pf) and incubated in the dark for 20 min at 4 °C. Then, samples were dehydrated by consecutive addition of 30%, 50%, 70%, 80%, and 90% ethanol and then kept in 100% (*v*/*v*) ethanol (Sigma-Aldrich). Coverslips were dried using the critical-point drying method and then metalized. Cells were observed in a scanning electron microscope (SU8010, Hitachi, Chiyoda, Japan) and digital images were acquired.

### 2.8. Multiparametric Flow Cytometry Assays

In the current study, multiparametric flow cytometry was used to immunophenotype moDCs and T cell subsets.

The supernatant of each well, containing nonadherent cells (lymphocytes), was collected and centrifuged (400× *g*, 10 min, 4 °C), and the pellet was resuspended in 200 μL PBS-Pf. Then, cells were incubated for 20 min at 4 °C in the dark. After incubation, cells were centrifuged (500× *g*, 10 min, 4 °C) and the pellet was washed with 1× PBS and resuspended in 100 μL of 1× PBS with 2% (*v*/*v*) FBS (PBS-FBS).

Afterward, the wells were washed with warm 1× PBS, followed by warm 1× PBS with 2 mM EDTA (PBS-EDTA) and then incubated with 500 μL of warm PBS-EDTA per well for 15 min at 37 °C in a humidified atmosphere containing 5% CO_2_. To promote detachment, cells were gently resuspended in the wells, collected, and washed with 1× PBS. Then, 500 μL of warm PBS-EDTA was added per well for a second 15 min incubation, after which the cells were collected and washed. This procedure leads to complete detachment of adherent cells (moDCs) while preserving membrane receptors. Therefore, the cell suspension was collected and centrifuged (400× *g*, 10 min, 4 °C). The pellet was resuspended in 200 μL PBS-Pf and then incubated in the dark for 20 min at 4 °C. Then, cells were centrifuged (500× *g*, 10 min, 4 °C), the pellet was washed with 1× PBS (500× *g*, 10 min, 4 °C), and the cells were resuspended in 100 μL of PBS-FBS.

Anti-CD3, anti-CD4, anti-CD8, anti-CD25, anti-CD44, and anti-CD62L monoclonal antibodies directly conjugated with fluorochromes were added to the lymphocytes, and anti-MHCI and anti-MHCII directly conjugated monoclonal antibodies were mixed with moDCs (Table S1). Cells were incubated in the dark for 30 min at 4 °C and then centrifuged. The pellet was washed with 100 μL of 1× PBS to ensure the removal of any residual antibodies that might bind non-specifically to cells. Afterward, the pellet was resuspended in 200 μL of 1× PBS.

Cells previously labeled with anti-CD3, anti-CD4, anti-CD8, and anti-CD25 monoclonal antibodies directly conjugated were intracellularly labeled with anti-FoxP3 monoclonal antibody directly conjugated. For intracellular labeling, cells were resuspended in 200 μL of permeabilization buffer [1× PBS 1% (*v*/*v*) FBS, 0.1% (*w*/*v*) sodium azide (NaN_3_, Sigma-Aldrich), 0.5% (*v*/*v*) Triton-X (Sigma-Aldrich)] at pH 7.4–7.6 and incubated for 20 min in the dark at room temperature. This buffer creates pores in both cytoplasmic and nuclear membranes, enabling the antibody to access and bind the nuclear factor FoxP3. Then, the cell suspension was centrifuged (500× *g*, 5 min, 4 °C), and the pellet was resuspended in the residual volume, labeled with FoxP3 monoclonal antibody, and incubated for 30 min in the dark at room temperature. Cells were washed with 100 μL 1× PBS (500× *g*, 5 min, room temperature), and the pellet was resuspended in 200 μL of 1× PBS.

Cell acquisition was performed on a CytoFLEX flow cytometer (Beckman Coulter), and each sample was performed in duplicate.

To ensure accurate readings, compensation was performed taking into consideration the calibration of the flow cytometer, background fluorescence, spillover spread from single-stained cells, and fluorescence minus one (FMO) control. Unstained cells were then used to define the gatings [35].

For each sample, 10,000 gated events were acquired, and the gating strategy was defined based on the compensation adjustment performed. In all samples, a first FSC-H vs. SSC-H gate was established to exclude any cell debris or unwanted cells. Doublet cells were eliminated through FSC-H vs. FSC-A gating.

To analyze the moDCs, a gating was performed to obtain MHCI^+^, MHCII^+^, and MHCI^+^MHCII^+^ cells. To examine T cell subsets, a more complex gating strategy was performed. First, a CD3^+^ gating was performed to target T cell populations (CD3 is T cell-specific). Afterward, a second gate was conducted to obtain CD3^+^CD4^+^ (helper T cells) or CD3^+^CD8^+^ (cytotoxic T cells) cell subsets. Then, CD4^+^ or CD8^+^ T cells that exhibited memory (CD62L and CD44) or regulatory (CD25 and FoxP3) phenotype were also assessed. Data analysis was performed on CytExpert 2.4 software (Beckman Coulter).

### 2.9. Reverse Transcription Real-Time Quantitative PCR

The gene expression of innate immune receptors and immune mediators of lymphocytes and moDCs in co-culture was evaluated by RT-qPCR, as well as resting lymphocytes and moDCs. RNA was extracted, followed by cDNA synthesis, and then RT-qPCR was performed for Toll-like receptors (TLR) 2, TLR4, and TLR9, IL-1β, IL-4, IL-10, IL-12p40 (a heterodimer of IL-12), interferon (IFN)-γ, tumor necrosis factor (TNF)-α, and tumor growth factor (TGF)-β. β-actin was used as a housekeeping gene. All samples were analyzed in duplicate for each gene.

To lyse the cells while preserving the RNA, lymphocytes and moDCs were stored in NR buffer (Nzytech, Lisbon, Portugal) supplemented with β-mercapto-ethanol (Sigma-Aldrich), and RNA extraction was performed using NZY Total RNA Isolation kit (Nzytech) according to the manufacturer’s instructions and then eluted in 40 μL of RNase-free water. RNA samples were processed into cDNA synthesis using the NZY First-strand cDNA Synthesis Kit (Nzytech) according to the manufacturer’s instructions. For each sample, a mix was prepared with 10 μL of NZYRT 2× Master Mix, 2 μL of NZYRT Enzyme Mix, and 8 μL of extracted RNA. PCR amplification was performed according to the manufacturer’s guidance conditions. After cDNA synthesis, RNase H was added to degrade any RNA–cDNA hybrids. cDNA was therefore diluted in 40 μL of RNase-free water.

Primers were selected based on the published works of several authors [20,36,37,38,39,40,41,42] (Table S2) and, for the determination of gene copies, plasmid DNA containing the insert fragment of each gene (standards) was produced as previously described by Rodrigues et al. [34].

Serial dilutions of standards were amplified to generate calibration curves, which allow the absolute quantification of each gene copy number. Plasmid DNA was quantified in a Nanodrop^®^ 1000 spectrophotometer (Thermo Fisher Scientific) and, for each gene, 1:5 serial dilutions were prepared in ultra-pure water, ranging from 250 pg.μL^−1^ to 0.016 pg.μL^−1^.

For every sample, standard, or blank, 20 μL of the real-time PCR mix reaction was prepared with 2 μL of sample cDNA or with standard dilution or ultra-pure water, 10 μL of iTaq™ SsoAdvanced™ Univ SYBR^®^ Green Supermix (Bio-Rad, Hercules, CA, USA), 0.15 μL of 20 pmol.μL^−1^ forward primer solution, 0.15 μL of 20 pmol.μL^−1^ reverse primer solution, and 7.7 μL of ultra-pure water. Amplification was performed using a Bio-Rad CFX Maestro PCR System thermal cycler (Bio-Rad) with the following conditions: 5 min at 95 °C for complete DNA denaturation, 40 cycles of 30 s at 95 °C and 30 s at the specific annealing temperature of each gene for annealing and extension, and 90 cycles of 10 s at the starting temperature of 50 °C with an increment of 0.5 °C for each cycle to obtain the melting curve. The fluorescence levels of each sample were analyzed in real-time by the thermal cycler, and the number of gene copies was calculated by comparing with the calibration curves.

β-actin was used as an internal control for gene expression, as it maintains a constant expression despite sample conditions. The number of copies of each gene was normalized to β-actin to minimize differences in the quantities of the initial cDNA of the sample. Therefore, the number of copies of each gene in each sample is expressed by the number of gene copies per 1000 copies of β-actin.

### 2.10. Data Analysis

The Zeta potential of EVs was compared with the negative control using Student’s *t*-test, with a significance level of 5%. Kruskal–Wallis test followed by Dunn’s multiple comparisons test at 5% significance level was used to compare protein immune reactivity. The data from at least six dogs and two replicates per sample were compared across different conditions. Since the data were not normally distributed, as indicated by the Kolmogorov-Smirnov test, the non-parametric Wilcoxon signed-rank test was used to assess the difference between paired groups. Statistical significance was demonstrated by a 5% significance level (*p* < 0.05), and statistical analysis and graphics were performed using GraphPad Prism 10 software (GraphPad Software Inc., Boston, MA, USA).

## 3. Results

### 3.1. Size Profile and Antigenic Reactivity of L. infantum-Derived EVs

**Figure 2:**
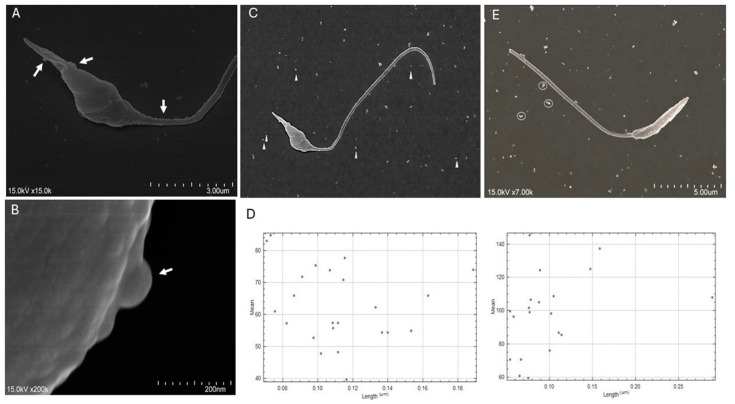
Scanning Electron Microscopy images of *L. infantum* promastigotes budding EVs. EVs budding from *L. infantum* promastigotes are indicated in panels (**A**,**B**) (arrows). Free EVs are shown in panel (**C**) (arrowheads), and EV aggregates are highlighted in panel (**E**) (circles). Panels (**D**) present the size distribution of EVs derived from panels (**C**,**E**), respectively, that exhibit a distinct signal from the background. This figure is displayed at the highest available resolution.

Scanning electron microscopy images acquired at the highest achievable resolution enabled the observation *L. infantum* promastigotes releasing EVs (Figure 2A) as well as a detailed visualization of EVs budding from a promastigote (Figure 2B). Furthermore, EVs’ size distribution (Figure 2D,E) obtained from EVs promastigotes surrounding promastigotes (Figure 2C,E) demonstrates the presence of EVs compatible with exosomes and micro-vesicles.

**Figure 3:**
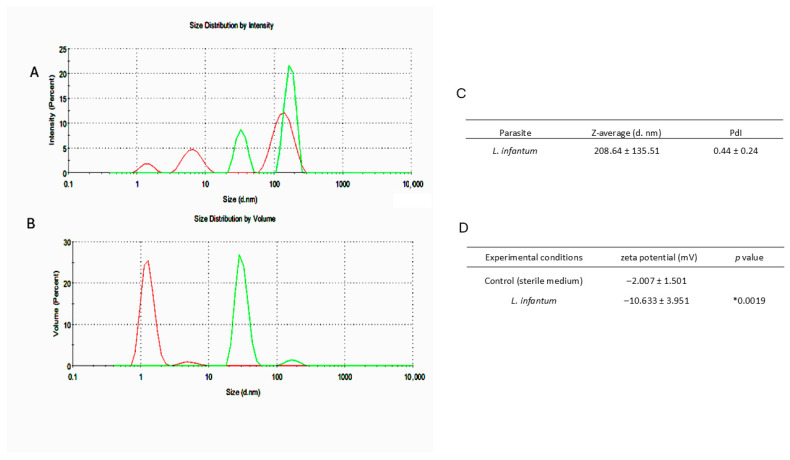
Characterization of *L. infantum* isolated extracellular vesicles by DLS and ELS. Graphical representation of intensity (**A**) and volume (**B**) of purified *L. infantum* derived EVs (green) and the control of EVs’ extraction method (red). Z-average, polydispersity index (PdI) (**C**), and Zeta potential (**D**) of the isolated EVs are indicated. Results are presented as mean ± standard deviation of three independent isolates (*n* = 3). * Indicates statistically significant differences.

DLS analysis showed a bimodal size distribution with a dominant peak ranging from 120–200 nm alongside a secondary peak at 50 nm (Figure 3A), which is consistent with a suspension enriched in exosomes and micro-vesicles, corroborating the findings previously described (Figure 2). In contrast, the negative control appears to include small particles (<10 nm) with no significant signal in the EVs’ size range (Figure 3B), which may indicate the precipitation of proteins from the culture medium. This confirms that the larger vesicles are derived from parasites and not from medium components. EVs derived from *L. infantum* promastigotes are polydisperse, with a Z-average diameter of 208.64 ± 135.51 nm and a polydispersity index of 0.44 ± 0.24 (Figure 3C), indicating a high degree of heterogeneity, which is characteristic of biological EVs’ isolates. Moreover, *L. infantum*-derived EVs exhibited a negative zeta potential (ζ = −10.633 ± 3.951 mV, Figure 3D), which is consistent with the presence of lipid bilayer membranes, since cellular membranes are generally negatively charged. In contrast, the negative control showed a higher zeta potential (ζ = −2.007 ± 1.501 mV), which is significantly different from EVs suspension (*p* = 0.0019), supporting the absence of membrane structures. The negative charge of EVs likely contributes to colloidal suspension by reducing EVs’ aggregation, increasing the stability of EVs’ suspension, and preventing nonspecific fusion, while facilitating receptor-mediated interactions with target cells. Moreover, negative control does not exhibit proteolytic activity and does not stimulate immune cells.

**Figure 4:**
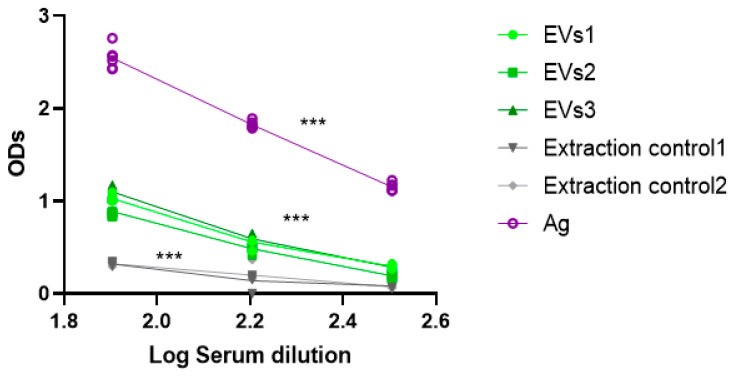
Specific immune reactivity of *L. infantum* EVs. Antigen reactivity of three independent EVs’ isolates (EVs1, EVs2, and EVs3) was analysed by ELISA using *L. infantum*-positive canine serum. Soluble *L. infantum* antigen (Ag) was included as a positive control along with two independent negative controls (extraction control 1 and extraction control 2). *** (*p* < 0.001) indicates statistically significant differences. ODs—Optical densities.

To demonstrate the presence of *Leishmania* proteins in the isolated EVs, an in-house ELISA assay using a commercial *L. infantum* positive serum was used. The assay showed a clear dilution-dependent signal for AgT, validating the assay performance. Three independent EVs’ isolates (EV1–EV3) were assessed, each demonstrating a consistent dilution-dependent reactivity above the negative control (Figure 4). Although the EVs’ associated signals were significantly lower than Ag reactivity (*p* < 0.001), they are clearly distinguished from the background generated by the negative control (*p* < 0.001), indicating that EVs contain *L. infantum* antigens/proteins that are specifically recognized by canine serum. The negative control exhibited a low signal consistent with the precipitation of culture medium components, as also suggested by the DLS results. However, these precipitates are expected to generate a nonspecific ELISA background. Therefore, the low background observed in negative controls supports the evidence that EVs’ signal is not an artifact of the isolation procedure but rather reflects *L. infantum*-derived EVs. Overall, the data support the reproductivity of the isolation method, as well as the presence of antigenic active *L. infantum* proteins in EVs, which are consistently recognized by the serum from a *L. infantum*-infected dog.

### 3.2. Co-Cultured Cells Remain Viable and Retain Functionality

**Figure 5:**
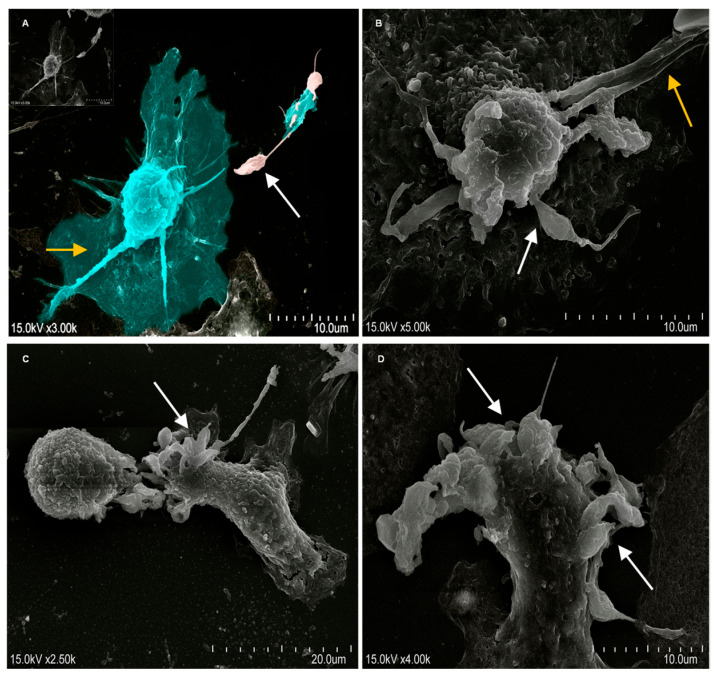
Interaction of moDCs with *L. infantum* promastigotes. Two (**A**,**B**) and four hours (**C**,**D**) of moDCs incubation with *L. infantum* promastigotes were observed by scanning electron microscopy, and digital images were acquired. Yellow arrows indicate moDCs’ cytoplasmic projections (dendrites), and white arrows indicate *L. infantum* promastigotes interacting with moDCs. (**A**)—Image edited to enhance the visualization details between moDCs and *Leishmania* promastigotes.

*L. infantum* promastigotes started interacting with the cytoplasmic membrane of moDCs after 2 h (Figure 5A,B) of incubation, and after 4 h (Figure 5C,D), the parasites became internalized. These results indicate that moDCs not only established contact with the parasites but also were able to phagocytose them, thereby acquiring parasite antigens for processing.

As the interaction between DCs and lymphocytes is key for initiating an adaptive immune response, the viability of moDCs and lymphocytes was assessed separately after 72 h in co-culture.

**Figure 6:**
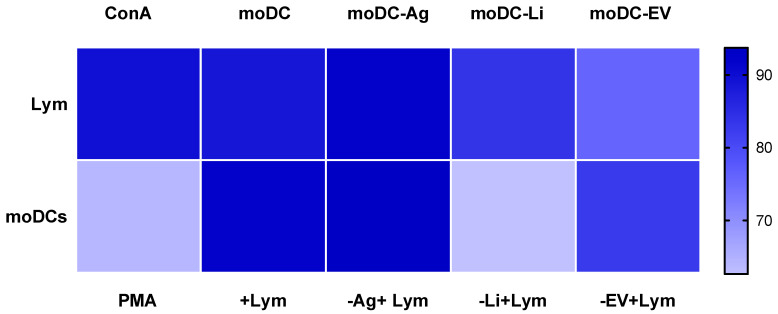
Viability of lymphocytes and autologous moDCs in co-culture. Cell viability was evaluated using multiparametric flow cytometry with annexin and propidium iodide, and the viability index was estimated by excluding both early and late apoptotic cells. The median of six samples (*n* = 6) is represented by the heat map generated by GraphPad Prism 10. Lym- lymphocytes, ConA- Lymphocytes stimulated by Concanavalin A, moDC-Ag-moDCs primed by parasite antigen, moDC-Li-moDCs infected by *L. infantum*, moDC-EV-moDCs primed by EVs, PMA-moDCs stimulated by PMA, +Lym-moDCs in co-culture with lymphocytes, -Ag+Lym-moDCs primed by parasite antigen in co-culture with lymphocytes, -Li+Lym-moDCs infected by *L. infantum* in co-culture with lymphocytes, and -EV+Lym-moDCs primed by EVs in co-culture with lymphocytes.

In co-culture, both lymphocytes and moDCs showed a high viability index (~90%). Lymphocytes stimulated by ConA, as well as lymphocytes co-cultured with Ag-primed or infected moDCs, maintained high viability (~88%). Ag- or EV-primed moDCs in co-culture were also viable (~84%). However, a slight decrease in viability was found in lymphocytes (~76%) co-cultured with EVs-primed moDCs, in moDCs stimulated by PMA (~64%), and in infected moDCs (~63%) co-cultured with lymphocytes (Figure 6). Taken together, the proportion of proapoptotic and apoptotic moDCs and lymphocytes in coculture was around 10%. Lymphocytes cocultured with infected moDCs or EVs-primed moDCs showed increased apoptotic cells (~20–25%). The highest levels of apoptosis were found in moDCs stimulated by PMA or in cocultured infected moDCs (~40%). In contrast, cocultures of Ag-stimulated moDCs displayed lower apoptosis (~6%).

Although *L. infantum* promastigotes and EVs appear to reduce the viability of moDCs and lymphocytes, respectively, the overall results indicate that most of the co-cultured cells remain viable and reactive.

### 3.3. In the Co-Culture System EVs-Primed moDCs Highly Upregulate TLR2, TLR4, and TLR9 and Generate a Mix of Pro- and Anti-Inflammatory Cytokines

Pattern recognition receptors (PRRs) are innate immune receptors that are signalized by conserved molecular patterns present in pathogens. Toll-like receptors (TLRs), a type of PRRs, recognize specific ligands and trigger a downstream signaling pathway, driving the host immune response. Therefore, to examine the activation of moDCs in co-culture with autologous lymphocytes, the gene expression of cell surface TLR2 and TLR4, and the endosomal TLR9 was assessed in moDCs infected by *L. infantum* promastigotes or primed by Ag or EVs.

**Figure 7:**
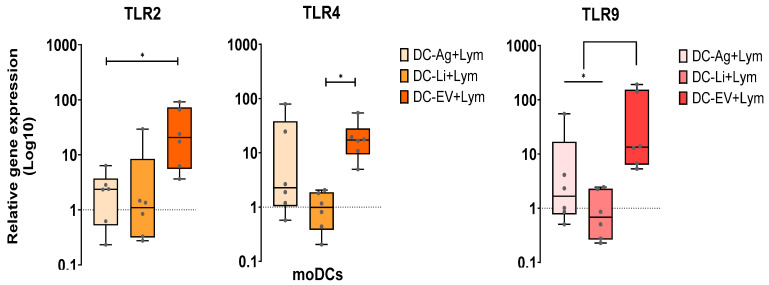
Relative gene expression of surface TLR2 and TLR4 and endocytic TLR9 by loaded moDCs co-cultured with autologous lymphocytes. moDCs infected by *L. infantum* (DC-Li+Lym) or stimulated by Ag (moDC-Ag+Lym), or EVs (DC-EV+Lym) were normalized to co-cultures with unloaded moDCs. The dotted line indicates the TLR basal levels. Data from six independently analyzed samples (*n* = 6) performed in duplicate are represented by boxplots, showing all points, interquartile ranges, median, minimum, and maximum values. The dotted line indicates the basal gene expression levels. The nonparametric Wilcoxon test was used for statistical comparisons. * (*p* < 0.05) indicates statistically significant differences.

TLR2, TLR4, and TLR9 gene expression were significantly upregulated in EV-primed moDCs co-cultured with lymphocytes, compared to infected moDCs (*p* = 0.0312) for TLR4 and TLR9, and to Ag-primed moDCs for TLR2 and TLR9. In contrast, infected moDCs showed low gene expression of TLR2, TLR4, and TLR9 in co-cultured conditions, and were similar to unloaded moDCs co-cultured with lymphocytes. Moreover, primed moDCs presented upregulation of these innate receptors when compared with unloaded moDCs in co-culture (Figure 7).

Therefore, these results suggest that in the presence of lymphocytes, Ag and EVs induce moDCs to upregulate surface TLR2 and TLR4, as well as endosomal TLR9, while *L. infantum* parasites have no impact on the analyzed TLRs. Ag and EVs may have ligands to induce the overexpression of TLR2, TLR4, and TLR9.

Cytokines are small proteins secreted by cells that are involved in cell signaling. DCs release specific cytokines and are susceptible to cytokine-mediated activation. Therefore, the gene expression of pro-inflammatory IL-1β, TNF-α, and IL-12p40 by loaded moDCs in co-culture, as well as the anti-inflammatory IL-10, was evaluated and normalized with unloaded moDCs in co-culture.

**Figure 8:**
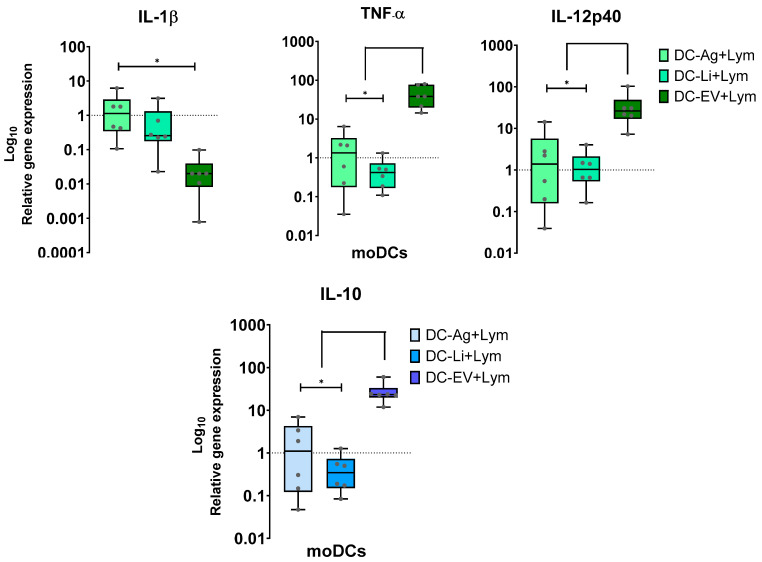
Generation of pro-inflammatory and anti-inflammatory cytokines by loaded moDCs co-cultured with autologous lymphocytes. Gene expression of IL-1β, IL-12p40, IL-10, and TNF-α by moDCs infected by *L. infantum* (DC-Li+Lym) or stimulated by Ag (moDC-Ag+Lym) or EVs (DC-EV+Lym) was normalized to co-cultures with unloaded moDCs. Data from six independently analyzed samples (*n* = 6) performed in duplicate are represented by boxplots, showing all points, interquartile ranges, minimum, maximum, and median values. The dotted line represents the basal levels of cytokines. The nonparametric Wilcoxon test was used for statistical comparisons. * (*p* < 0.05) indicates statistically significant differences.

In co-cultures, EVs loaded moDCs showed downregulation of IL-1β, which was significantly different from Ag-primed moDCs (*p* = 0.0321). In contrast, EVs primed moDCs showed a marked upregulation of IL-12p40 and TNF-α (*p* = 0.032) when co-cultured with lymphocytes. IL-10 upregulation was also found in EVs-primed moDCs, exhibiting a significant difference compared to Ag-primed moDCs and infected moDCs (*p* = 0.0321). *L. infantum* parasites and Ag had no significant impact on IL-12p40, TNF-α, and IL-10 gene expression, which remained similar to unloaded moDCs (Figure 8).

Thus, in moDC-lymphocyte co-culture, EVs favor the generation of anti-inflammatory IL-10 by moDCs, as well as pro-inflammatory IL-12 and TNF-α, while inhibiting the generation of pro-inflammatory IL-1β. This promotes the establishment of a microenvironment with a mix of pro- and anti-inflammatory immune mediators. Meanwhile, moDCs primed with Ag or infected with *L. infantum* parasites can generate IL-1β while maintaining baseline levels of IL-12, TNF-α, and IL-10.

Taken together, these results suggest that moDCs primed by EVs become activated and have the potential to elicit a balanced immune response, restraining excessive inflammation.

### 3.4. In the Co-Culture System L. infantum-EVs Induces the Predomination of MHCI^+^ and MHCII^+^ moDCs

To evaluate whether loaded moDCs may present antigens to T lymphocytes, the frequency of MHCI^+^ and MHCII^+^ moDCs was assessed by multiparametric flow cytometry. Compared to unloaded moDCs co-cultured with lymphocytes, infected moDCs and EVs-primed moDCs were able to cross-present antigens, while EVs and Ag-primed moDCs could present parasite antigens to helper T cells.

**Figure 9:**
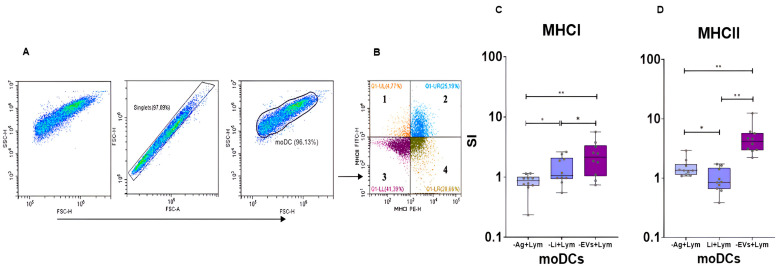
MHCI and MHCII surface expression in loaded moDCs co-cultured with lymphocytes. After excluding cell debris, unwanted cells (dead cells and lymphocytes), and doublets (**A**), a dot plot was generated (**B**) to determine distinct moDCs subsets based on the surface expression of MHCI and MHCII. 1—MHCI^−^MHCII^+^ moDCs, 2—MHCI^+^MHCII^+^ moDCs, 3—MHCI^−^MHCII^−^ moDCs, and 4—MHCI^+^MHCII^−^. MHCI (**C**) and MHCII (**D**) SI were evaluated in moDCs infected with *L. infantum* promastigotes (-Li+Lym), primed by antigens (-Ag+Lym) or extracellular vesicles (-EVs+Lym). Unprimed moDCs were used as negative controls. Data from ten independently analyzed samples (*n* = 10) are represented by boxplots, which show all points, interquartile ranges, minimum, maximum, and median values. The nonparametric Wilcoxon test was used for statistical comparisons. * (*p* < 0.05) and ** (*p* < 0.01) indicate statistically significant differences.

moDCs primed by EVs showed the highest stimulation index (SI) of MHCI and MHCII, which was statistically different from Ag-primed moDCs (*p*_MHCI_ = 0.0059, *p*_MHCII_ < 0.002) and infected moDCs (*p*_MHCI_ = 0.0195; *p*_MHCII_ < 0.002). Although with a lower SI (average 2), MHCI^+^ moDCs infected by *L. infantum* promastigotes also presented a significant increase (*p* = 0.0059) when compared with Ag-primed moDCs. In contrast, MHCII^+^ moDCs infected by *L. infantum* promastigotes showed a decrease (*p* = 0.0488) in comparison with Ag-primed moDCs (Figure 9).

In co-culture, moDCs primed with *L. infantum* EVs predominantly exhibited MHCI and MHCII phenotypes, suggesting possible antigen presentation to both helper T cells (CD4^+^ T cells) and cytotoxic T cells (CD8^+^ T cells), whereas *L. infantum*-infected moDCs appear to evade antigen presentation to helper T cells.

### 3.5. In the Co-Cultured System L. infantum Ag-Primed moDCs Induce the Expansion of CD8^+^ T Cells

To characterize how *L. infantum* promastigotes, Ag or EVs loaded moDCs affect T cell subpopulations, CD4^+^ and CD8^+^ expression by T cells were evaluated by multiparametric flow cytometry. After a CD3^+^ (T cell-specific marker) cell gating, the frequency of CD4^+^ and CD8^+^ T cells was determined.

**Figure 10:**
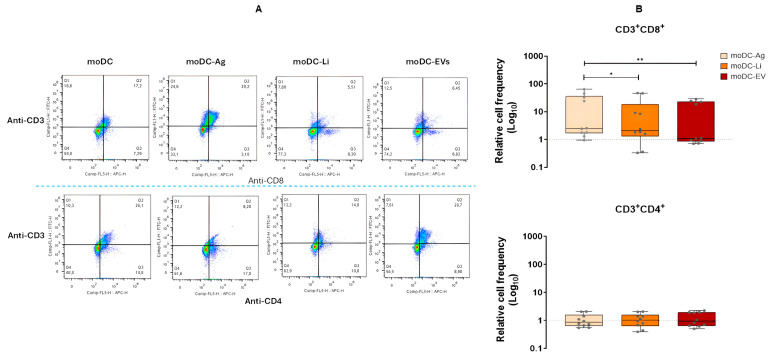
CD8^+^ and CD4^+^ T cell subpopulations co-cultured with loaded moDCs. After excluding cell debris, doublets, and unwanted cells (dead cells and moDCs), CD3^+^ cells were gated to define the overall population and then to obtain CD3^+^CD8^+^ and CD3^+^CD4^+^ dot plots (**A**) in co-cultures with *L. infantum* promastigotes (moCD-Li), antigens (moDC-Ag), or extracellular vesicles (moDC-EV) loaded moDCs. The relative frequency of CD8^+^ and CD4^+^ T cells (**B**) in co-cultures with *L. infantum* promastigotes (moCD-Li), antigens (moDC-Ag), or extracellular vesicles (moDC-EV) loaded moDCs was evaluated and normalized to co-cultures with unloaded moDCs (moDC). Data from 10 independently analyzed samples (*n* = 10) are represented by box plot graphs, which show all points, interquartile ranges, minimum, maximum, and median values. The dotted line indicates the basal levels of T cell subpopulations. The nonparametric Wilcoxon test was used for statistical comparisons. * (*p* < 0.05) and ** (*p* < 0.01) indicate statistically significant differences.

After normalization to T lymphocytes co-cultured with unloaded moDCs, the CD4^+^ T cell subset from co-cultures with Ag-primed moDCs, infected moDCs, or EVs-primed moDCs showed values identical to those of co-cultures with unloaded moDCs. In contrast, there was a marked expansion of the CD8^+^ T cell subpopulation that was higher in co-cultures with Ag-primed moDCs. This expansion was significantly different from co-cultures with infected moDCs (*p* = 0.0488) or with EVs-primed moDCs (*p* = 0.002) (Figure 10).

Overall, these findings indicate that in co-culture, *L. infantum*-infected moDCs and Ag-primed moDCs promote an imbalance in T cell expansion, favoring the immune response towards cytotoxic T cells. Furthermore, EVs primed moDCs have a minimal influence on helper T cells, also favoring cytotoxic T cell predominance, thereby contributing to an imbalance between these two T cell subpopulations.

### 3.6. In the Co-Cultured System EVs-Primed moDCs Trigger Lymphocytes to Generate Pro- and Anti-Inflammatory Cytokines

Immune mediators, like cytokines, are small proteins released by lymphocytes that play a crucial role in activating and regulating the host’s immune defense. Therefore, the effect of loaded moDCs on lymphocyte gene expression of pro- (IL-12p40, IFN-γ, and TNF-α) and anti-inflammatory cytokines (IL-4, TGF-β, and IL-10) was estimated.

**Figure 11:**
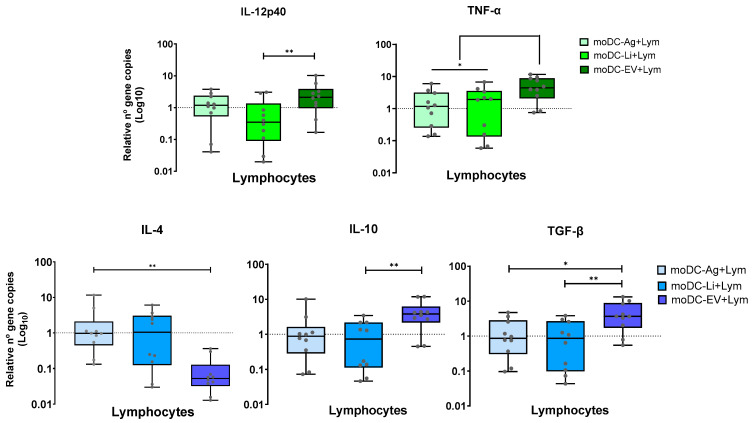
Cytokine gene expression by lymphocytes in co-culture with loaded moDCs. Gene expression of IL-12p40, TNF-α, IL-4, IL-10, and TGF-β by lymphocytes in co-culture with *L. infantum* promastigotes (Li), antigens (Ag), or extracellular vesicles (EV) loaded moDCs was normalized to co-cultures with unloaded moDCs. Data from seven independently analyzed samples (*n* = 7) performed in duplicate are represented by boxplots, showing all points, interquartile ranges, minimum, maximum, and median values. The dotted line represents the basal levels of cytokines. The nonparametric Wilcoxon test was used for statistical comparisons. * (*p* < 0.05) and ** (*p* < 0.01) indicate statistically significant differences.

After normalization to unstimulated co-cultures, lymphocytes co-cultured with EVs primed moDCs showed a marked upregulation of IL-12p40 (*p* = 0.002) when compared with infected moDCs and of TNF-α in comparison with Ag-primed moDCs (*p* = 0.0195) and infected moDCs (*p* = 0.0137). However, IFN-γ gene expression in co-cultured lymphocytes was basal. Regarding anti-inflammatory cytokines, significant upregulation of TGF-β was found in co-cultures of moDCs primed by EVs compared to co-cultures of infected moDCs (*p* = 0.002) and Ag-primed moDCs (*p* = 0.0195). Moreover, when compared with moDCs primed by parasite Ag, IL-10 also showed significant upregulation (*p* = 0.002). In contrast, co-cultures of EVs primed moDCs showed a significant IL-4 downregulation (*p* = 0.0019) compared with co-cultured moDCs primed by Ag (Figure 11).

Therefore, moDCs primed by EVs can trigger lymphocytes to generate both pro- and anti-inflammatory cytokines, except for IFN-γ, that were unaffected, and IL-4, which was suppressed.

### 3.7. In the Co-Culture System EVs-Primed moDCs Favor an Unconventional Regulatory Immune Response

Regulatory T cells (Tregs) are essential for maintaining immune homeostasis during the host immune response. Therefore, the frequency of CD4^+^ and CD8^+^ Treg in co-culture with *L. infantum*-infected moDCs or moDCs primed by Ag or EVs was assessed. Treg cells exhibit a CD25^+^FoxP3^+^ phenotype, while cells lacking CD25^−^ and FoxP3^−^ are considered naïve T cells. On the other hand, antigenically activated T cells (or effector cells) are typically CD25^+^FoxP3^−^, whereas T cells lacking CD25 and expressing FoxP3^+^ are recognized as non-conventional regulatory T cells.

**Figure 12:**
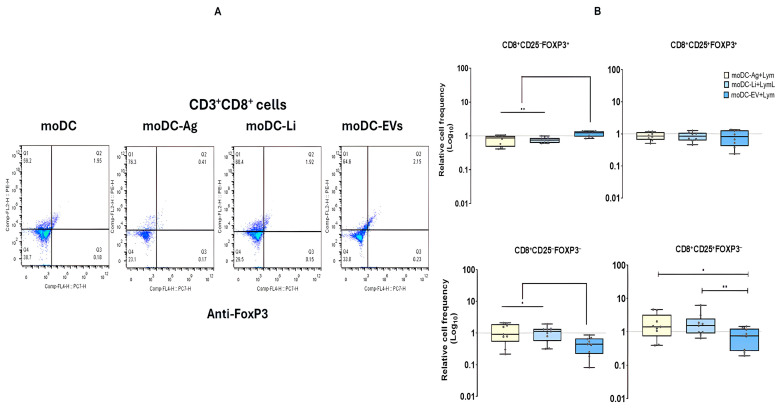
CD8^+^ T cell subsets with CD25 and FoxP3 phenotype in co-culture with loaded moDCs. From CD3^+^CD8^+^ cell subpopulation co-cultured with *L. infantum* promastigotes (moCD-Li+Lym), antigens (moDC-Ag+Lym), or extracellular vesicles (moDC-EV+Lym) loaded moDCs, CD25^+^ cells and FoxP3^+^ cells were gated and dot-plots were generated (**A**). The relative frequency of CD3^+^CD8^+^CD25^−^FoxP3^+^, CD3^+^CD8^+^CD25^+^FoxP3^+^, CD3^+^CD8^+^CD25^−^FoxP3^−^, and CD3^+^CD8^+^CD25^+^FoxP3^−^ was normalized to the respective subset from co-cultures with unloaded moDCs. Data from eight independently analyzed samples (*n* = 8) are represented by box plot graphs, which show all points, interquartile ranges, minimum, maximum, and median values (**B**). The dotted line indicates the basal levels of T cell subsets. The nonparametric Wilcoxon test was used for statistical comparisons. * (*p* < 0.05) and ** (*p* < 0.01) indicate statistically significant differences.

CD4^+^ T cell subsets normalized to the respective lymphocyte cell subsets of unloaded moDCs co-cultures did not show alterations, remaining at steady-state levels. In contrast, CD8^+^ T cells exhibited alterations in the levels of various subsets. EVs primed moDCs promoted significant contraction of CD8^+^CD25^+^FoxP3^−^ and CD8^+^CD25^−^FoxP3^−^ T cell subsets when compared with co-cultures of infected moDCs (*p*_CD25−FoxP3−_= 0.0195; *p*_CD25+FoxP3−_ = 0.008) and Ag-primed moDCs (*p*_CD25−FoxP3−_ = 0.0039; *p*_CD25+FoxP3−_ = 0.00273). However, EV-primed moDCs also caused a significant expansion of CD8^+^CD25^−^FoxP3^+^ cells in comparison with infected moDCs and Ag-primed moDCs (*p* = 0.0078). In co-cultures with loaded moDCs, CD8^+^CD25^+^FoxP3^+^ T cell subsets remained at basal levels (Figure 12).

Therefore, loaded moDCs do not favor important changes in regulatory CD4^+^ or CD8^+^ T cells. However, EVs-primed moDCs appear to exhibit a pattern of restraining the effector CD8^+^ T cell subset, which can favor parasite survival, not inducing proliferation of effector T cells. On the other hand, the expansion of non-conventional regulatory T cell subsets, along with the reduction of naïve T cells, can impact the effector immune response, suppressing immune activation. Overall, co-cultures of EVs primed moDCs seem to control specific T cell responses, contributing to parasite survival.

### 3.8. In the Co-Culture System moDCs Primed by Ag or EVs Trigger the Establishment of Central Memory T Cells

Cellular memory has a significant impact on infection outcome as it provides a faster and more accurate response upon reinfection. The effect of loaded moDCs on the levels of memory T cells was evaluated. The frequency of memory CD4^+^ T cell subsets after co-culture with moDCs infected with *L. infantum* promastigotes or Ag or EVs primed moDCs was characterized by multiparametric flow cytometry, and the results were normalized to lymphocytes in co-culture with unloaded moDCs.

Regarding CD8^+^ T cell subsets, a significant increase of CD44^+^CD62L^+^ T cell subset (*p* = 0.0078) was verified in co-cultures with moDCs primed by EVs in comparison with co-cultures with infected moDCs, as well as the expansion of CD44^+^CD62L^−^ T cell subset. Furthermore, CD44^−^CD62L^−^ and CD44^+^CD62L^−^ T cells also showed slight increases in co-cultures with infected moDCs or Ag-primed moDCs (Figure 12A,B).

**Figure 13:**
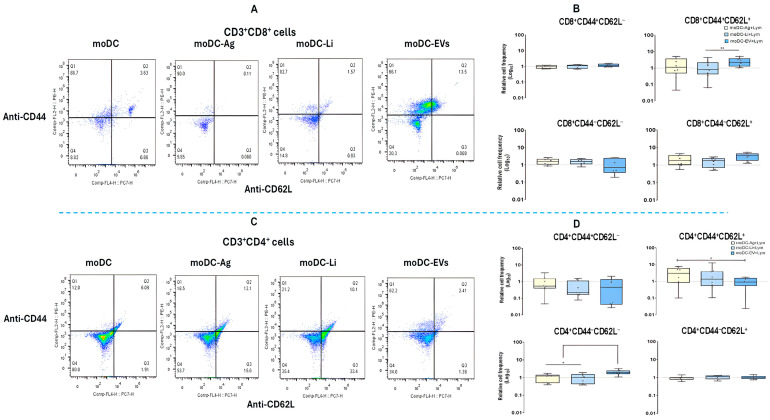
CD8^+^ and CD4^+^ T cell subsets expressing CD44 and CD62L phenotype in co-culture with loaded moDCs. From CD8^+^ T cells and CD4^+^ T cells co-cultured with moDCs loaded with *L infantum* (Li), antigens (Ag), or extracellular vesicles (EV), CD44^+^ and CD62L^+^ cells were gated, and dot plots were generated (**A,C**). The relative frequency of CD3^+^CD8^+^CD25^−^FoxP3^+^, CD3^+^CD8^+^CD25^+^FoxP3^+^, CD3^+^CD8^+^CD25^−^FoxP3^−^, CD3^+^CD8^+^CD25^+^FoxP3^−^ (**B**), CD3^+^CD4^+^CD25^−^FoxP3^+^, CD3^+^CD4^+^CD25^+^FoxP3^+^, CD3^+^CD4^+^CD25^−^FoxP3^−^, and CD3^+^CD4^+^CD25^+^FoxP3^−^ (**D**) was normalized to the respective subset from co-cultures with unloaded moDCs. Data from eight independently analyzed samples (*n* = 8) are represented by box plot graphs, which show all points, interquartile ranges, minimum, maximum, and median values. The dotted line indicates the basal levels of T cell subsets. The nonparametric Wilcoxon test was used for statistical comparisons. * (*p* < 0.05) and ** (*p* < 0.01) indicates statistically significant differences.

On the other hand, EVs-primed moDCs induced a significant expansion of CD4^+^CD44^−^CD62L^−^ T cell subset in comparison with infected moDCs (*p* = 0.0195) and Ag-primed moDCs (*p* = 0.0177). Besides, Ag-primed moDCs caused a significant expansion of CD4^+^CD44^+^CD62L^+^ T cell subset (*p* = 0.0273) when compared with lymphocytes co-cultured with EVs-primed moDCs. However, in co-cultures with infected or primed moDCs, CD4^+^CD44^−^CD62L^+^ T cell subset was reduced, and the CD4^+^CD44^+^CD62L^−^ T cell subset was found to be similar to the respective cell subset co-cultured with unloaded moDCs (Figure 13C,D).

Thus, in co-culture, EVs-primed moDCs caused a boost of CD4^+^CD44^+^CD62L^−^ T cells, which represent naïve cells, and CD8^+^ (CD44^+^CD62L^+^) central memory T cells (T_CM_), which play a crucial role in long-term immune protection. Moreover, Ag-primed moDCs trigger the expansion of the CD4^+^ T_CM_ cell subset. *L. infantum-infected* moDCs and Ag-primed moDCs favor the expansion of CD8^+^ naïve T cells (CD44^−^CD62L^−^) and CD8^+^CD44^+^CD62L^+^ T cell subset, which appear to be transitory cells that circulate between lymphoid tissues, playing a role in immune surveillance.

## 4. Discussion

Leishmaniasis is an ancient parasitic disease with diverse clinical manifestations affecting humans and wild and domestic animals [43]. The control of this disease remains elusive due to the complex interplay of biological, environmental, and socioeconomic factors. Environmental and climatic changes, along with evolving social dynamics, have altered the epidemiological aspects of this disease, contributing to its emergence in new regions, highlighting the urgent need for innovative strategies capable of reducing the global burden of this disease. Therefore, in this context, dendritic cells have emerged as a promising tool that can open new avenues for host-specific immune modulation and the potential to induce long-lasting protection.

The present study explored the immune programming of T cells by autologous DCs exposed to *L. infantum* parasites or primed by parasite soluble Ag or EVs, which can be used to predict disease outcome. These findings may contribute to innovative control strategies against leishmaniasis within the One Health approach, ultimately supporting the development of effective immune therapeutics, reducing severe disease, and efficient prophylactic tools to prevent infection.

The high viability of cells underscores the effectiveness of the coculture system employed in this study, which supports cellular interactions between moDCs and lymphocytes and demonstrates their capacity to respond to stimuli without undergoing significant apoptosis. However, the increased apoptosis found in moDCs conditioned by PMA or EVs likely reflects stress or high cellular activity, which can be associated with the substantial upregulation of innate receptors and the generation of immune mediators, or the increased expression of MHC molecules.

To start the crosstalk with lymphocytes, DCs must recognize highly conserved molecular patterns of pathogens, such as nucleic acids, proteins, and lipids, through PRRs, such as TLRs. Upon activation, these innate sensors trigger downstream signaling cascades that can lead to cytokine production, which helps shape the effector T cell response. In parallel, DCs process captured parasite antigens, which are presented to T cells through MHC molecules. This combination of *Leishmania* antigenic presentation and cytokine signaling bridges innate parasite detection with adaptive immune response, determining the immune response.

In *Leishmania* infection, the most studied TLRs are TLR2, TLR4, and TLR9. TLR2-activated pathways may result in host-protective or disease-exacerbating immune responses, depending on the species of *Leishmania*. In *L. infantum* infection, TLR2 and TLR9 activation lead to a host-protective immune response [44] that ensures parasite control. A study by Li and collaborators [45] demonstrated that in DCs, TLR2 signaling induces IL-1β secretion. In contrast, TLR4 signaling plays a regulatory role by limiting parasite-specific immune response and controlling chronic inflammation, which is associated with the absence of clinical signs. A recent study revealed that the activation of the TLR4 pathway can downregulate the immune response against *L*. *infantum* infection, reducing Th1 cell activity to prevent excessive immune response [46]. The findings of the current study suggest that the upregulation of TLR2, TLR4, and TLR9 in moDCs may indicate enhanced recognition of parasite proteins and nucleic acids carried by *L. infantum*-derived EVs, implying activation of associated sensing pathways. Furthermore, it appears that parasite antigens are also recognized by these receptors, albeit to a lesser extent. Sacramento and colleagues [47] reported that TLR9 recognizes CpG DNA motifs of *Leishmania* spp., while Schleicher et al. [48] had demonstrated that *L. infantum* can activate myeloid DCs through TLR9, leading to the production of IL-12p40. The current study findings suggest that *L. infantum* EVs activate the endosomal TLR9 pathway of moDCs, leading to the generation of IL-12p40. Moreover, these findings also indicate that both *L. infantum* parasites and Ag promote a slight generation of IL-12p40. Altogether, these data support the understanding that parasite EVs interact with the moDC membrane, are internalized, and deliver parasite DNA, triggering the TLR9 signaling cascade. This TLR9 signaling promotes IL-12p40 gene expression, potentially contributing to Th1 cell activation. A previous study by our group [24] demonstrated that EVs derived from *Leishmania* spp. rapidly adhere to mouse macrophages, influencing their immune activation. In contrast, *L. infantum*-derived EVs do not induce moDCs to generate the proinflammatory IL-1β, but parasites and Ag highly promote their generation. In a previous study, it was demonstrated that, in bone marrow DCs, TLR2 signaling induces IL-1β secretion [45]. Pro IL-1β is synthesized early in response to pathogen sensing, and its posterior activation relies on inflammasome assembly. Once activated, IL-1β enhances the expression of adhesion molecules on vascular endothelial cells, facilitating the migration of immune cells to the site of infection, thus initiating the inflammatory response. Signaling of TLR4 pathways can result in TNF-α synthesis, while TLR2 activation is associated with IL-10 induction. In the context of *L. braziliensis* infection, TLR4 signaling leads to TNF-α production [49]. On the other hand, the inhibition of TLR2 signaling by *L. donovani* parasites reduces IL-10 synthesis [50], highlighting its regulatory role. In the current study, unlike moDCs primed by EVs in co-culture with autologous lymphocytes, which highly promote the generation of proinflammatory TNF-α and regulatory IL-10, moDCs infected with *L. infantum* or primed by parasite Ag exhibited low induction of these cytokines. The robust cytokine generation by EV-primed moDCs is consistent with a significant upregulation of TLR4 and TLR2 found in these cells. Therefore, TLR activation of moDCs by *L. infantum*-derived EVs favors a hybrid immune response, characterized by pro-inflammatory and regulatory cytokines, thereby preventing extreme polarization toward a pro-inflammatory immune response.

In dogs, the outcome of CanL is primarily determined by the cell-mediated immune response, in which T lymphocytes play a significant role. The outcome of this disease depends on the balance between a Th1 or Th2 immune response. Th1 effector cells can produce IL-12, IFN-γ, and TNF-α, which enhance the activity of effector CD8^+^ T cells and trigger a respiratory burst in infected macrophages. The synthesis of reactive oxygen species and nitric oxide production is crucial for parasite elimination [51]. However, *Leishmania* parasites have developed mechanisms to interfere with the IFN-γ signaling required for iNOS synthesis, consequently preventing effective parasite lysis [52]. In contrast, a predominant Th2 response promotes the release of anti-inflammatory cytokines, such as IL-4 and IL-10, along with the stimulation of the humoral immune response, which contributes to parasite immune evasion, facilitating the establishment of infection and leading to disease progression [51]. However, many dogs exhibited a mix of Th1/Th2 immune responses associated with variable clinical signs [53].

The findings of the current study suggest that EV-primed moDCs can present antigens to T cells by increasing the frequency of MHCI^+^ and MHCII^+^ moDCs, thereby directing the activation of cytotoxic (CD8^+^) and helper (CD4^+^) T cells within a favorable cytokine environment. However, the expansion of these moDC subsets does not align with the increase in either T cell subpopulations. This discrepancy is likely due to the generation of regulatory immune mediators, such as IL-10 and TGF-β, which may suppress effector T cells. Thus, although EV-primed moDCs are viable and maintain their antigen-presenting capacity, there may be a dysregulation between moDCs activation and T lymphocyte expansion. In contrast, the increase in T cells is associated with a contraction or slight expansion of MHCI^+^ moDCs and MHCII^+^ moDCs subsets previously exposed to *L. infantum* parasite or primed by Ag. This response may be linked to the induction of IL-1β, a cytokine that can favor a pro-inflammatory environment, supporting T cell expansion [54,55,56].

To eliminate *Leishmania* parasites, a strong pro-inflammatory response must be accompanied by the release of IFN-γ and TNF-α, which can suppress Treg cells or make effector cells resistant to suppression mediated by Treg cells. However, during chronic infection, the persistence of pro-inflammatory cytokines can lead to self-tissue damage. Therefore, Tregs are recruited to infection sites to limit the consequences of chronic inflammation and promote homeostasis, although this allows parasite survival [57,58]. On the other hand, the increase in IL-10 secretion inhibits the proliferation of helper and cytotoxic T cells, which reduces IFN-γ production and enhances activation of Treg cells [59]. These cells can be characterized as natural Treg, which develop in the thymus, or Treg cells that differentiate in peripheral lymphoid tissues and are considered as Treg cells induced in the periphery. Regardless of their origin, Treg cells are characterized by the presentation of a CD25^+^FoxP3^+^ phenotype. The induction of nuclear transcription factor FoxP3 by the regulatory cytokine TGF-β is essential for CD25^−^ T cells to acquire suppressive functions [27,28]. The CD25^−^FoxP3^+^ T cell subset constitutes a peripheric reservoir of non-conventional Treg cells that can be recruited to the CD25^+^ pool as part of the homeostatic response [29]. Although less widely explored, the CD25^+^FoxP3^−^ T cell subset appears to lie at the interface between the states of activation and regulation. Surface expression of CD25, which is the α chain of IL2 receptor, indicates an activated phenotype. On the other hand, CD25^−^FoxP3^−^ T cells can be naïve cells or quiescent memory cells.

The findings of the current study demonstrate that loaded moDCs did not induce alterations in the levels of CD4^+^ T cell subsets expressing CD25 or FoxP3, nor promote a conventional CD8^+^ or CD4^+^ T regulatory response. According to Mahnke and collaborators [57], only mature DCs can stimulate T cell proliferation, whereas steady-state DCs trigger Treg cells. Rather than promote immune tolerance, parasite or Ag-loaded moDCs favor the induction of a cytotoxic T cell response. Interestingly, moDCs primed with *L. infantum* EVs can shape cytotoxic T cell phenotype towards a non-conventional regulatory profile. This shift can be associated with the generation of regulatory cytokines by both moDCs (IL-10) and lymphocytes (TGF-β and IL-10). Moreover, the balance of pro-inflammatory and regulatory cytokines modulated by EVs highlights their potential for application in the development of an innovative prophylactic strategy.

In American cutaneous leishmaniasis, there is an increase in the frequency of both T_CM_ and T_EM_ cells, especially within the CD8^+^ T cell population [60]. Furthermore, another study demonstrates that infection with *L. major* leads to the differentiation of T_CM_ cells that remain in the absence of parasites. Upon reinfection, these memory cells rapidly become effector T cells, contributing to a protective immune response [61]. In *L. infantum*-infected BALB/c mice, liver resident T cells differentiate into CD4^+^ and CD8^+^ T_CM_ cell subsets after antigen-specific recall. However, following conventional antileishmanial treatment, there is a marked expansion of the CD4^+^ T_EM_ cell subset [42].

In the current study, moDCs primed by *L. infantum* Ag exhibited a decline in the naïve CD4^+^ T cell population, suggesting that these moDCs are effective antigen-presenting cells that drive the differentiation and expansion of CD4^+^ T_CM_ cells. This quiescent cell subset homes in the secondary lymphoid organs, where it retains the memory of a previous encounter with parasite antigens. Upon re-exposure to the same antigen that triggered the differentiation, these cells can rapidly proliferate and differentiate into effector CD4^+^ T cells, thereby mounting a robust secondary immune response. In contrast, the sustained availability of naïve and intermediate CD8^+^ T cell subsets suggests a balanced differentiation of CD8^+^ T cells driven by antigen presentation via moDCs either infected with *L. infantum* or primed with Ag. This is further supported by a small expansion of CD8^+^ T cells expressing CD25. Moreover, moDCs primed by EVs promote the rise of intermediate cells and decline of naïve CD4^+^ T cell subsets, without a corresponding expansion of memory cells, which indicates limited antigenic stimulation and establishment of a steady-state long-term immunity. Furthermore, EV-primed moDCs favored the expansion of memory CD8^+^ T cells, especially CD8^+^ T_CM_ cells, along with an increase in naïve cells. These findings point to a tightly regulated activation of cytotoxic T cells, which is consistent with the low levels of effector CD8^+^ T cells and the small increase of non-conventional regulatory CD8^+^ T cells.

Despite the potential restraining of effector response, moDCs primed by EVs support the expansion of memory T cell subsets, which can promote long-lasting protective immunity mediated by cytotoxic T cells. This cytokine-balanced T cell response minimizes inflammation while establishing a pool of memory CD8^+^T cells, which is crucial for controlling *Leishmania*-infected cells. Thus, T cell immune modulation by EV-pulsed moDCs reflects an immune profile preferred for an effective prophylactic strategy against *Leishmania* infection. Despite its relevance for the design of effective prophylactic and therapeutic interventions, studies addressing long-term immune memory of CanL are scarce, especially regarding the differentiation and maintenance of memory T cell pools.

Therefore, moDCs loaded by *Leishmania*-derived EVs can elicit an effective adaptive immune response specifically targeting *Leishmania* antigens, while minimizing off-target immune effects. Moreover, personalized DCs presenting parasite antigens to autologous T cells enable the precise recognition of *Leishmania* epitopes, which can vary between individuals, minimizing the risk of anergic or suboptimal immune responses. This approach opens new avenues for the development of prophylactic or immunotherapeutic precision vaccines based on cell-based strategies, which represent a potential pathway toward innovative translation interventions for leishmaniasis control. Although the present in vitro study highlights the EVs’ potential for developing strategies to control leishmaniasis, further ex vivo research using tissue-resident DCs, as well as in vivo studies, are essential to validate and extend these findings.

## 5. Conclusions

**Figure 14:**
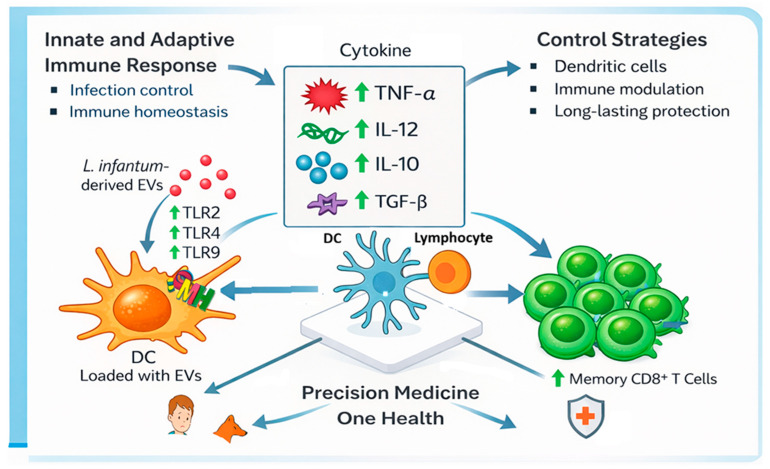
Schematic illustration highlighting the main findings of the current study and its future implications. Immune activation of lymphocytes was directed by autologous dendritic cells loaded with EVs derived from *L. infantum* parasites, favoring infection control and immune homeostasis as well as the expansion of specific memory T cells. These insights may pave the way to the development of innovative precision medicine strategies for leishmaniasis management aligned with the One Health approach.

The current study demonstrates for the first time that the interaction between canine T lymphocytes and autologous moDCs primed by *L. infantum* EVs promoted a balanced inflammatory and regulatory response, which favors infection control while maintaining homeostasis (Figure 14). Given that EV-primed moDCs can activate and direct T lymphocyte response, the potential of EVs to shape cellular immunity could be translated into realistic applications, such as personalized prophylactic strategies or immunotherapies. While further studies are needed, these findings highlight a promising first step toward the development of precision medicine strategies for leishmaniasis, aligned with the integrative One Health perspective.

## Data Availability

The original contributions presented in this study are included in the article/supplementary material. Further inquiries can be directed to the corresponding author.

## Supplementary Materials

The following supporting information can be downloaded at: https://www.mdpi.com/article/10.3390/cells15100919/s1, Table S1: Panel of moDCs and lymphocyte monoclonal antibodies used in multiparametric flow cytometry assays. title. Table S2: Primer sequences used for gene expression of innate receptors and immune modulators.

## Abbreviations

The following abbreviations are used in this manuscript:

Ag	*L. infantum* soluble antigen
APCs	Antigen-presenting cells
CanL	Canine leishmaniasis
ConA	Concanavalin A
CSF	Colony-stimulating factor
DCs	Dendritic cells
DMSO	Dimethyl sulfoxide
EVs	Extracellular vesicles
hiFBS	Heat-inactivated fetal bovine serum
IFN	Interferon
IL	Interleukin
MHC	Major histocompatibility complex
MHCI	MHC class I molecules
MHCII	MHC class II molecules
moDCs	Monocyte-derived DCs
PBMCs	Peripheral blood mononuclear cells
PBS	Phosphate buffer saline
Pen-Strep	Penicillin-streptomycin
PI	Propidium iodide
PMA	Phorbol myristate acetate
RPMI	Roswell Park Memorial Institute 1640 medium
RT-qPCR	Reverse transcription real-time quantitative PCR
SCHN	Schneider’s Drosophila medium
NaCl	Sodium chloride
T_CM_	Central memory T cells
TCRs	T cell receptors
T_EM_	Effector memory T cells
Th1	Type 1 T helper cells
TLR	Toll-like receptors
Treg	Regulatory T cells
