# Supplementary material for: Exploring Autologous Dendritic Cells for T Cell Modulation: A Step Towards Personalized Medicine in Leishmaniasis
Source: Cells. 2026 May 18;15(10):919. doi: 10.3390/cells15100919 (PMC13204335; doi:10.3390/cells15100919)
Supplement: Supplementary file 1 [file cells-15-00919-s001.zip › revised_DC_Tcell_TableS1 .pdf]

Article title: Exploring Autologous Dendritic Cells for T Cell Modulation: A Step Towards Personalized Medicine in Leishmaniasis

Journal name: Cells

Autor names: Mafalda Meunier, Ana Valério-Bolas, Armanda Rodrigues, Flávia Frois-Martins, Rui Ferreira, Inês Cardoso, Marta Monteiro, Joana Palma-Marques, Manuela Carvalheiro, Telmo Nunes, Wilson T Antunes, Graça Alexandre-Pires, Isabel Pereira da Fonseca, Gabriela Santos-Gomes

**Laser lines**	**488 nm**					**640 nm**			
**Emission filters**	**525/40**		**585/42**			**665/20**		**780/60**	
**Biomarker**	CD3e	MHCII	CD44	FoxP3	HLA ABC (MHCI)	CD4	CD8a	CD62L	CD25
**Fluorochrome**	FICT		PE		RPE	APC		APC-Cyanine7	PE-Cyanine7
**Brightness**	2		5		5	5		2	4
**Antibody**	Mouse anti-dog	Rat anti-dog	Mouse anti-dog	Rat anti-dog	Rat anti-mouse*	Rat anti-dog		Rat anti-mouse*	Mouse anti-dog
**Clone**	CA17.2A12	YKIX334.2	MEM-263	FJK-16s	W&/32	YCATE55.9	YCATE55.9	MEL-14	P4A10
**Company**	Invitrogen	Bio-Rad	Invitrogen		Bio-Rad	Invitrogen		Invitrogen	Invitrogen
**Concentration ($mg.mL^{-1}$)**	0.2		0.1	0.2	0.1	0.1	0.5	0.1	0.1

Table S1. Panel of lymphocyte monoclonal antibodies used in multiparametric flow cytometry assays. Specifications for anti-MHCI. anti-MHCII, anti-CD3e, anti-CD4, anti-CD8a, anti-CD44, anti-CD62L, anti-CD25 and anti-FoxP3 monoclonal antibodies are described. Fluorochrome brightness is rated on a scale from 1 to 5 (with 5 indicating the brightest).

* Sequence similarity was assessed using BLAST results with a query coverage greater than 85%.
