# Supplementary material for: Exploring Autologous Dendritic Cells for T Cell Modulation: A Step Towards Personalized Medicine in Leishmaniasis
Source: Cells. 2026 May 18;15(10):919. doi: 10.3390/cells15100919 (PMC13204335; doi:10.3390/cells15100919)
Supplement: Supplementary file 1 [file cells-15-00919-s001.zip › revised_DC_Tcell_TableS2.pdf]

Article title: Exploring Autologous Dendritic Cells for T Cell Modulation: A Step Towards Personalized Medicine in Leishmaniasis

Journal name: Cells

Autor names: Mafalda Meunier, Ana Valério-Bolas, Armanda Rodrigues, Flávia Frois-Martins, Rui Ferreira, Inês Cardoso, Marta Monteiro, Joana Palma-Marques, Manuela Carvalheiro Telmo Nunes, Wilson T Antunes, Graça Alexandre-Pires, Isabel Pereira da Fonseca, Gabriela Santos-Gomes

Gene	Primer sequence	Reference	Fragment size (bp)	T_{AN} (ºC)
**β-Actin**	Fw-5'ACGGAGCGTGGCTACAGC 3'<br>Rv-5' TCCTTGATGTCACGCACGA 3'	Sauter et al., 2005	61	60.5
**IL-1β**	Fw-5' CCATGAGGTGCGTGGATAC 3'<br>Rv-5' TGTGCAAGGGTGTGTTTCAT 3'	Peters et al., 2005	80	60.5
**IL-4**	Fw-5' CATCCTCACAGCGAGAAACG 3'<br>Rv-5' CCTTATCGCTTGTGTTCTTTGGA 3'	Huang et al., 2008	83	50
**IL-10**	Fw-5' CAAGCCCTGTCGGAGATGAT 3'<br>Rv-5' CTTGATGTCTGGGTCGTGGTT 3'	Yu et al., 2010	78	54
**IL-12p40**	Fw-5' CAGCAGAGAGGGTCAGAGGG 3'<br>Rv-5' ACGACCTCGATGGGTAGGC 3'	Peters et al., 2005	109	58
**IFN-γ**	Fw-5' TCAACCCCTTCTCGCCACT 3'<br>Rv-5' GCTGCCTACTTGGTCCCTGA 3'	Menezes-Souza et al., 2011	113	60
**TNF-α**	Fw-5' AATCATCTTCTCGAACCCCAAG 3'<br>Rv-5' GGAGCTGCCCCTCAGCTT 3'	Sauter et al., 2005	75	57
**TGF-β**	Fw-5' CAGAATGGCTGTCCTTTGATGTC 3'<br>Rv-5' AGGCGAAAGCCCTCGACTT 3'	Huang et al., 2008	79	60
**TLR2**	Fw-5' AATCCCCCGTTCAAGTGTG 3'<br>Rv-5' ATGGTTTTGCGGCTCTTCTC 3'	Ishii et al., 2006	101	61
**TLR4**	Fw-5' AGAGGATTTCCCCATTGGAC 3'<br>Rv-5' ACGCAGGTAGCTTGAAGGAA3'	Valério-Bolas, 2022	86	56
**TLR9**	Fw-5' ACCACATCATCACCCTGGCACCT 3'<br>Rv-5' CGGCGACAGTCCCACCCAC 3'	Rodrigues et al., 2017	82	64

Supplementary Table 3. Primer sequences. Forward (Fw) and reverse (Rv) primers, fragment size (bp), references, and annealing temperatures (T_{AN}) are provided for each gene.

References:

Peters I.R.; Helps C.R.; Calvert E.L.; Hall E.J.; Day M.J. Cytokine mRNA quantification in histologically normal canine duodenal mucosa by real-time RT-PCR. Vet Immunol Immunopathol. 2005, 103, 101-111. doi: 10.1016/j.vetimm.2004.08.020

Sauter S.N.; Allenspach K.; Gaschen F.; Gröne A.; Ontsouka E.; Blum J.W. Cytokine expression in an ex vivo culture system of duodenal samples from dogs with chronic enteropathies: modulation by probiotic bacteria. Domest Anim Endocrinol. 2005, 29, 605-622. doi: 10.1016/j.domaniend.2005.04.006

Ishii M.; Hashimoto M.; Oguma K.; Kano R.; Moritomo T.; Hasegawa A. Molecular cloning and tissue expression of canine Toll-like receptor 2 (TLR2). Vet Immunol Immunopathol. 2006, 110, 87-95. doi: 10.1016/j.vetimm.2005.09.007.

Huang Y.C.; Hung S.W-; Jan T.R.; Liao KW.; Cheng C.H.; Wang Y.S.; Chu R.M. CD5-low expression lymphocytes in canine peripheral blood show characteristics of natural killer cells. J Leukoc Biol. 2008, 84,1501-1510. doi: 10.1189/jlb.0408255

Yu D.H.; Noh D.H.; Song R.H.; Park J. Ethyl pyruvate downregulates tumor necrosis factor alpha and interleukin (IL)-6 and upregulates IL-10 in lipopolysaccharide-stimulated canine peripheral blood mononuclear cells. J Vet Med Sci. 2010, 72, 1379–1381. doi: 10.1292/jvms.09-0590

Menezes-Souza D, Guerra-Sá R, Carneiro CM, Vitoriano-Souza J, Giunchetti RC, Teixeira-Carvalho A, Silveira-Lemos D, Oliveira GC, Corrêa-Oliveira R, Reis AB. 2012. Higher expression of CCL2, CCL4, CCL5, CCL21, and CXCL8 chemokines in the skin associated with parasite density in canine visceral leishmaniasis. PLoS Negl Trop Dis. 6(4):e1566. doi: 10.1371/journal.pntd.0001566.

Rodrigues, A.; Claro, M.; Alexandre-Pires, G.; Santos-Mateus, D.; Martins, C.; Valério-Bolas, A.; Rafael-Fernandes, M.; Pereira, M.; da Fonseca, I.P.; Tomás,A.; et al. *Leishmania infantum* antigens modulate memory cell subsets of liver resident T lymphocyte. Immunobiology 2017, 222, 409–422. https://doi.org/10.1016/j.imbio.2016.08.009.

Valério-Bolas, A.; Meunier, M.; Palma-Marques, J.; Rodrigues, A.; Santos, A.M.; Nunes, T.; Ferreira, R.; Armada, A.; Alves, J.C.; Antunes, W.; et al. Exploiting *Leishmania* primed dendritic cells as potential immunomodulators of canine immune response. Cells 2024, 13, 445. https://doi.org/10.3390/cells13050445.
